# SARS-CoV-2 Harnesses Host Translational Shutoff and Autophagy To Optimize Virus Yields: the Role of the Envelope (E) Protein

**DOI:** 10.1128/spectrum.03707-22

**Published:** 2023-01-09

**Authors:** Hope Waisner, Brandon Grieshaber, Rabina Saud, Wyatt Henke, Edward B. Stephens, Maria Kalamvoki

**Affiliations:** a University of Kansas Medical Center, Department of Microbiology, Molecular Genetics, and Immunology, Kansas City, Kansas, USA; Indian Institute of Science Bangalore

**Keywords:** autophagy, protein synthesis, SARS-CoV-2 E protein, HSV-1 γ1 34.5 protein, ER stress, translation control, virus replication, HSV-1, PERK, SARS-CoV-2

## Abstract

The SARS-CoV-2 virion is composed of four structural proteins: spike (S), nucleocapsid (N), membrane (M), and envelope (E). E spans the membrane a single time and is the smallest, yet most enigmatic of the structural proteins. E is conserved among coronaviruses and has an essential role in virus-mediated pathogenesis. We found that ectopic expression of E had deleterious effects on the host cell as it activated stress responses, leading to LC3 lipidation and phosphorylation of the translation initiation factor eIF2α that resulted in host translational shutoff. During infection E is highly expressed, although only a small fraction is incorporated into virions, suggesting that E activity is regulated and harnessed by the virus to its benefit. Consistently, we found that proteins from heterologous viruses, such as the γ_1_ 34.5 protein of herpes simplex virus 1, prevented deleterious effects of E on the host cell and allowed for E protein accumulation. This observation prompted us to investigate whether other SARS-CoV-2 structural proteins regulate E. We found that the N and M proteins enabled E protein accumulation, whereas S did not. While γ_1_ 34.5 protein prevented deleterious effects of E on the host cells, it had a negative effect on SARS-CoV-2 replication. The negative effect of γ_1_ 34.5 was most likely associated with failure of SARS-CoV-2 to divert the translational machinery and with deregulation of autophagy. Overall, our data suggest that SARS-CoV-2 causes stress responses and subjugates these pathways, including host protein synthesis (phosphorylated eIF2α) and autophagy, to support optimal virus replication.

**IMPORTANCE** In late 2019, a new β-coronavirus, SARS-CoV-2, entered the human population causing a pandemic that has resulted in over 6 million deaths worldwide. Although closely related to SARS-CoV, the mechanisms of SARS-CoV-2 pathogenesis are not fully understood. We found that ectopic expression of the SARS-CoV-2 E protein had detrimental effects on the host cell, causing metabolic alterations, including shutoff of protein synthesis and mobilization of cellular resources through autophagy activation. Coexpression of E with viral proteins known to subvert host antiviral responses such as autophagy and translational inhibition, either from SARS-CoV-2 or from heterologous viruses, increased cell survival and E protein accumulation. However, such factors were found to negatively impact SARS-CoV-2 infection, as autophagy contributes to formation of viral membrane factories and translational control offers an advantage for viral gene expression. Overall, SARS-CoV-2 has evolved mechanisms to harness host functions that are essential for virus replication.

## INTRODUCTION

Compared with other highly pathogenic coronaviruses (CoVs), the mortality rate of SARS-CoV-2 is approximately 2% among unvaccinated individuals. This mortality rate, along with the lack of preexisting immunity, the fact that about 20% of infected individuals without preexisting immunity require medical attention, and the highly transmissible nature of the virus, has led to the disruption of normal activities worldwide for more than 2 years. Several highly effective vaccines have received use authorization, but the slow global vaccination rate and accumulation of adaptive mutations in different proteins of the virus, particularly the Spike protein, yield potential novel variants with different immunoevasion properties. Coronaviruses are enveloped viruses with a single-stranded, positive sense RNA genome. The coronavirus particle is composed of four structural proteins: nucleocapsid (N), membrane (M), envelope (E), and spike (S) (1). E is a small integral membrane protein that ranges from 75 to 106 aa (2). E protein localizes to the endoplasmic reticulum (ER), the ER-Golgi intermediate compartment (ERGIC) and the Golgi complex (3 to 7). The protein exists in different forms, including a monomeric form that potentially interacts with cellular proteins to alter the secretory machinery and to communicate signals, and a high-molecular weight homo-oligomer functioning in virion assembly (8, 9). In addition, a pentameric form of E protein is an ion channel (viroporin) with mild selectivity for cations that has been linked to virus pathogenesis (10–13).

The importance of E during SARS-CoV and SARS-CoV-2 infections is highlighted by the fact that viruses lacking the gene for E protein display significantly reduced virus yields due to aborted viral assembly that gives rise to immature virions with a strikingly aberrant morphology (5, 13, 14). For example, during infection with a mouse hepatitis virus (MHV) deleted of E, the virions display pinched and elongated, rather than spherical shapes and smaller, irregular-shaped plaques with jagged edges (15). How E protein facilitates virion morphogenesis remains unclear considering that only a small fraction of E is incorporated into the virions (16). A role of E in inducing membrane curvature has been proposed for MHV, perhaps associated with E homo-polymerization and its interactors, but a mechanism is currently unknown (6).

The role for the cation channel activity of E during SARS-CoV-2 infection is also unclear, although mutations within the transmembrane domain that inhibit the ion channel activity in SARS-CoV E are reversed by this virus (17). However, as most known mutations that impair the ion channel activity of E also impair E oligomerization, it is currently unknown if one or both properties of the protein are rescued (13). The transport of Ca^2+^ by SARS-CoV E has been correlated with inflammatory-mediated lung damage *in vivo*, highlighting the importance of E in viral pathogenesis (18, 19). The channel activity of E could also alter the secretory pathway or the luminal environment, leading to efficient trafficking of virions. Consistently, some of the proposed interactors of E are associated with ion transport and others with vacuoles and mitochondria, suggesting that E may participate in reorganizing membranes and the recruitment of lipid processing machineries at sites of virion assembly (4, 20–24).

Considering that E protein localizes in the ER-ERGIC-Golgi compartments and forms anion channel, we sought to determine the type of responses activated in cells ectopically expressing E. We found that E protein triggered ER-signaling pathways that led to phosphorylation of the translation initiation factor eIF-2α with a concomitant translational shutoff and LC3 lipidation. Both effects indicate that major metabolic alterations occur in cells expressing E that impact protein synthesis and potentially mobilize energy resources. We also found that E protein accumulation was restricted in cells ectopically expressing E protein. As a tool to further understand the functions of E, we used proteins from heterologous viruses known to prevent eIF-2α phosphorylation and LC3 lipidation and determined whether they could reverse the adverse effects of E on the host. The γ_1_ 34.5 protein of HSV-1 is known to prevent host translational shutoff during HSV-1 infection by recruiting the protein phosphatase 1α (PP1α) to dephosphorylate eIF-2α, which is phosphorylated by activated protein kinase R (PKR) following foreign RNA sensing (25–29). In addition, γ_1_ 34.5 protein inhibits autophagy by binding to the autophagy-inducing protein Beclin-1 that is downstream of activated PKR (29–32). Mutant HSV-1 viruses lacking the Beclin-1-interacting domain of γ_1_ 34.5 display reduced viral replication *in vitro* and *in vivo*, due to robust activation of autophagy (29–36). We found that γ_1_ 34.5 could reverse eIF-2α phosphorylation, but not LC3 lipidation induced by E, and enabled E protein accumulation.

An interesting observation was that HSV-1 γ_1_ 34.5 inhibited SARS-CoV-2 replication. One mechanism was through inhibition of the host translational shutoff by γ_1_ 34.5 that is imposed by the virus to gain translational advantage over the host. Consistent with this, an inhibitor of the PKR-like ER kinase PERK that inhibited phosphorylation of eIF-2α during SARS-CoV-2 infection caused a decrease in progeny virus production. Additionally, disruption of autophagy pathways by γ_1_ 34.5 during SARS-CoV-2 infection led to formation of aberrant vacuolar structures, most likely containing engulfed organelles, instead of forming viral membrane factories. Taken together, our data suggest that SARS-CoV-2 harnesses stress response pathways of the host for optimal progeny virus production.

## RESULTS

### The E protein of SARS-CoV-2 initiates autophagy and interferes with translation initiation.

**FIG 1:**
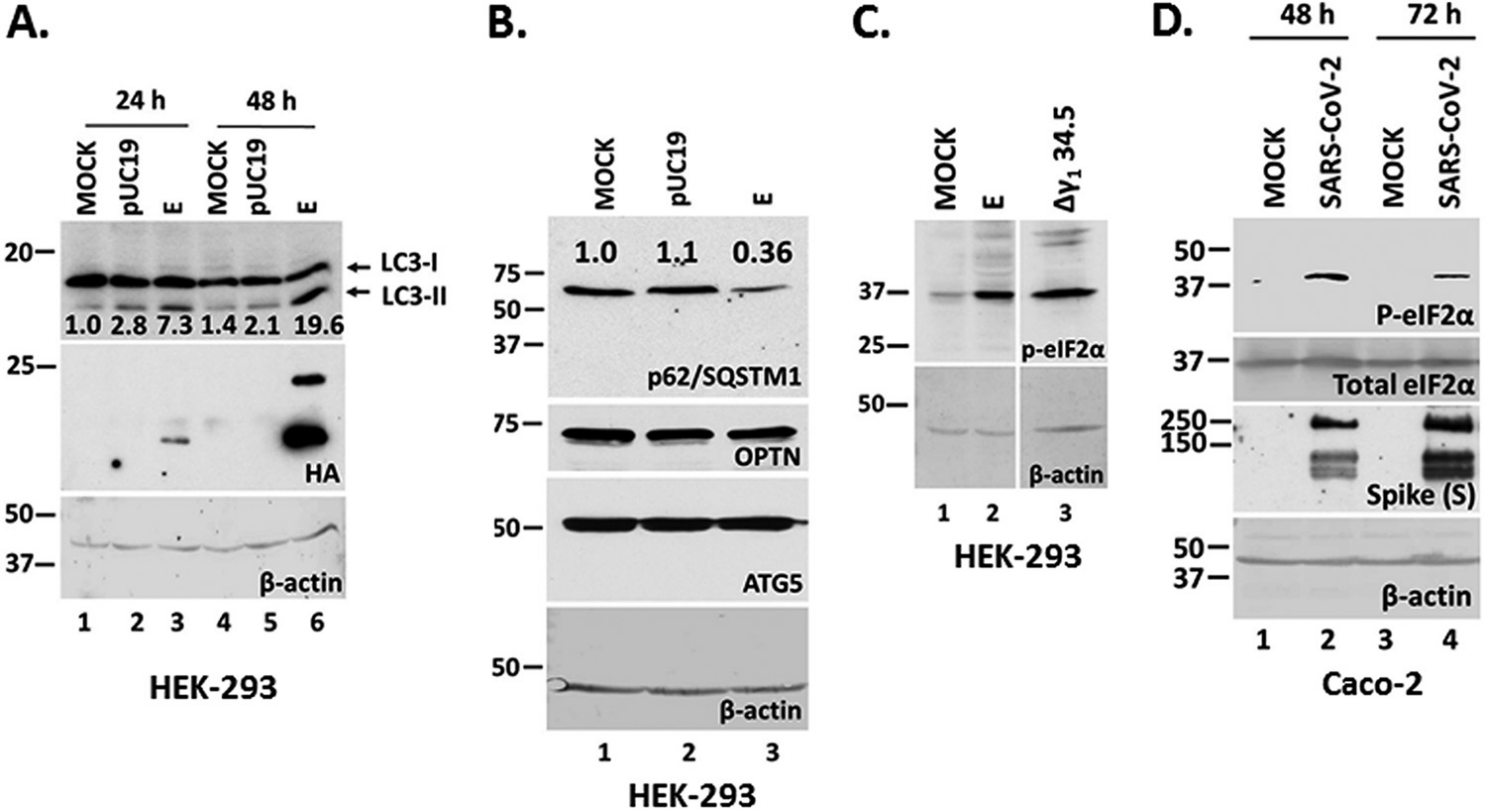
SARS-CoV-2 E expression causes LC3 lipidation, reduction in p62/SQSTM1 levels, and increased eIF-2α phosphorylation. (A) HEK-293 cells were either left untransfected, transfected with a control pUC19 plasmid, or an E-HA expressing plasmid. The cells were harvested at 24 and 48 h posttransfection and equal amounts of proteins were analyzed for LC3lipidation and E expression. The ratio of LC3-II/LC3-I is shown below. (B) Transfections were as in panel A. Equal amounts of proteins were analyzed for p62/SQSTM1, Optineurin and ATG5. (C) HEK-293 cells were transfected with an E-HA expressing plasmid or infected with a HSV-1 Δγ_1_ 34.5 virus (5 PFU/cell). The cells were harvested at 48 h posttransfection or at 14 h postinfection. Equal amounts of proteins were analyzed for p-eIF-2α. (D) Caco-2 cells were infected with SARS-CoV-2 (2 PFU/cell). The cells were harvested at 48 h and at 72 h postinfection and equal amounts of proteins were analyzed for p-eIF-2α and total eIF-2α. Spike served as a control for the infection. B-actin served as a loading control in panels A-D.

The E protein accumulates in the ER and ERGIC where it can form a channel with weak cation specificity, which may exhibit Ca^2+^ transport activity (3, 4, 7, 10, 11, 13). While only a small amount of E protein expressed during SARS-CoV-2 infection is incorporated into the virions, the protein appears to also induce membrane curvature, and participate in membrane scission (6, 16, 37, 38). Thus, we sought to determine ER signaling responses that may be activated by E expression. We found that ectopic expression of E in HEK-293 cells caused LC3 lipidation that was apparent by 48 h posttransfection and increased the LC3-II/LC3-I ratio (Fig. 1A). Moreover, we observed reduced p62/SQSTM1 accumulation that is indicative of autophagy activation (Fig. 1B). P62/SQSTM1 is an adaptor protein that sorts ubiquitinated cargo to autophagosomes for degradation and subsequently is degraded itself (39, 40). However, we did not observe changes in the levels of optineurin (OPTN) suggesting that mitophagy was not induced by E expression (Fig. 1B). Also, we did not observe changes in the levels of ATG5 protein (autophagy related 5), which along with ATG12 protein acts as an E1-activating enzyme during autophagy (Fig. 1B) (41). In addition, we tested whether E expression could trigger phosphorylation of the translation initiation factor eIF-2α, a modification that is usually observed when unfolded protein response (UPR) pathways are activated (42, 43). We observed that ectopic expression of E triggered accumulation of p-eIF-2α (Fig. 1C). As a control, HEK-293 cells were infected with an HSV-1 γ_1_ 34.5-null mutant, which cannot reverse phosphorylation of eIF-2α. Finally, following infection of Caco-2 cells with SARS-CoV-2 we observed accumulation of p-eIF-2α (Fig. 1D). We conclude that ectopic expression of E protein activates stress responses that lead to phosphorylation of eIF-2α and LC3 lipidation.

### The γ_1_ 34.5 protein of HSV-1 inhibited phosphorylation of eIF-2α but not LC3 lipidation induced by E expression.

**FIG 2:**
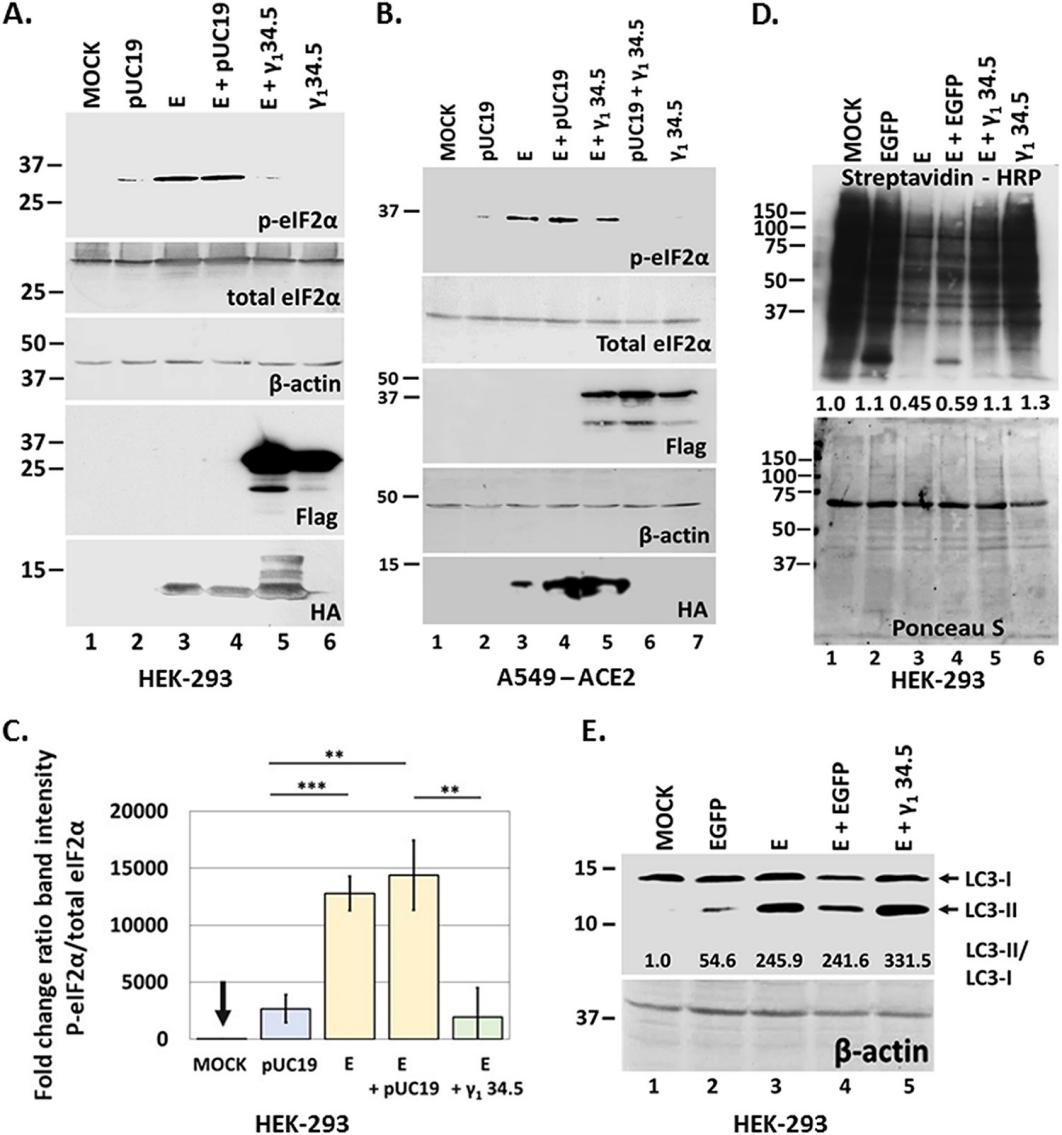
The γ_1_ 34.5 protein of HSV-1 prevents p-eIF-2α accumulation induced by E expression but not LC3 lipidation. (A) HEK-293 cells were either left untransfected, or transfected with the control plasmid pUC19, an E-HA expressing plasmid, cotransfected with E-HA and pUC19, E-HA and γ_1_ 34.5-expressing plasmids, or with a γ_1_ 34.5-expressing plasmid (Flag-tagged). The cells were harvested at 48 h posttransfection and equal amounts of proteins were analyzed for p-eIF-2α, total eIF-2α, E-HA, or γ_1_ 34.5 protein expression (Flag tagged). B-actin served as a loading control. (B) Experiment was as in panel A, but it was performed in A549-ACE2 cells. A549-ACE2 transfection efficiency was lower than in HEK-293 and that could account for some variability in the results. (C) Quantification of data from at least three independent experiments performed as in panel A. The fold change of p-eIF-2α/eIF-2α ratio of each sample compared with untransfected cells is depicted.(D) HEK-293 cells were either left untransfected or transfected with the control plasmid pLenti CMV GFP Puro expressing EGFP, an E-HA expressing plasmid, cotransfected with E-HA and pLenti CMV GFP Puro, E-HA and γ_1_ 34.5-expressing plasmids, or with a γ_1_ 34.5-expressing plasmid (Flag-tagged). At 46 h postinfection the cells were starved for 3 h in RPMI Medium 1640 without l-methionineand subsequently incubated with medium supplemented with Click-iT AHA reagent for 2 h. Click chemistry reaction to monitor nascent protein synthesis was performed as described in Materials and Methods. Both a Ponceau S staining of the membrane and the reaction of HRP with the ECL substrate (Pierce) are depicted. Quantification of total protein signal per sample relative to the signal of total proteins from untransfected cells, after normalization to the signal from Ponceau S staining is depicted. (E) Transfections in HEK-293 cells were as in panel A and samples were analyzed for LC3 lipidation with the ratio of LC3-II to LC3-I shown below. The pUC19 plasmid was replaced with pLenti CMV GFP Puro that expresses the control protein EGFP. B-actin served as a loading control.

The γ_1_ 34.5 protein of HSV-1 is known to prevent host translational shutoff (25–28, 33) and autophagy (30). To assess if γ_1_ 34.5 could reverse the effects of E protein, HEK-293 (Fig. 2A) or A549-ACE2 (Fig. 2B) cells were cotransfected with an E and a γ_1_ 34.5-expressing plasmid. Cells cotransfected with the E-expressing plasmid and an empty vector served as a control. Additional controls included cells transfected with the individual plasmids and untransfected cells. The cells were harvested at 48 h posttransfection and equal amounts of proteins were analyzed for p-eIF-2α. As shown in Fig. 2A-B, E expression triggered accumulation of p-eIF-2α that was blocked by the presence of γ_1_ 34.5. Quantification data of eIF-2α phosphorylation in E expressing HEK-293 cells in the presence or absence of γ_1_ 34. 5 protein, compared with controls, are depicted in Fig. 2C. The phosphorylation of eIF-2α due to E expression caused translational shutoff (Fig. 2D, lanes 3–4) that was partially reversed in the presence of γ_1_ 34.5 (Fig. 2D, lane 5). Quantification of the total protein signal per lane compared with the signal from untransfected cells following normalization to the respective Ponceau S signal is depicted (Fig. 2D). In a similar transfection assay, we tested if γ_1_ 34.5 could inhibit LC3 lipidation triggered by E expression. As shown in Fig. 2E, LC3 lipidation due to E expression was not reversed by γ_1_ 34.5. This is perhaps because γ_1_ 34.5 interferes with phagophore elongation through Beclin-1 binding, which does not necessarily interfere with LC3 lipidation. We conclude that γ_1_ 34.5 protein inhibits the phosphorylation of eIF-2α triggered by E expression but does not inhibit LC3 lipidation.

### The γ_1_ 34.5 protein of HSV-1 and the SARS-CoV-2 M and N proteins allowed for E protein accumulation.

**FIG 3:**
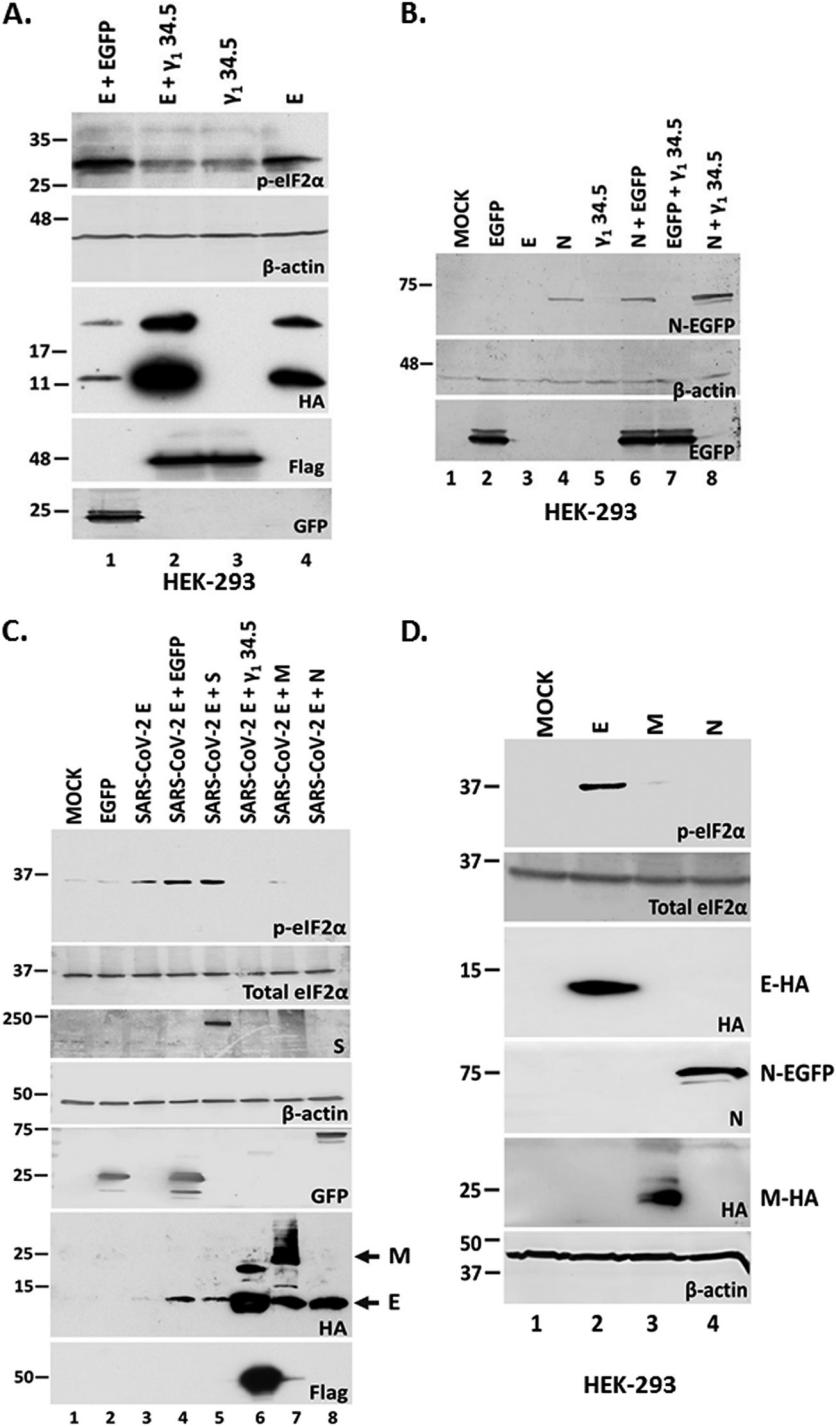
The γ_1_ 34.5 protein of HSV-1 and the SARS-CoV-2 M and N proteins resulted in E protein accumulation. (A) HEK-293 cells were transfected with an E-HA expressing plasmid, a γ_1_ 34.5-expressing plasmid (Flag-tagged), cotransfected with E-HA and γ_1_ 34.5-expressing plasmids, or E-HA and EGFP-expressing plasmids. The cells were harvested at 48 h posttransfection and equal amounts of proteins were analyzed for p-eIF-2α, E-HA, γ_1_ 34.5 expression (Flag tagged), or EGFP. B-actin served as a loading control. (B) Transfections were as in panel A. Cell lysates prepared at 48 h posttransfection were analyzed using a GFP antibody with β-actin serving as a loading control. (C–D) HEK-293 cells were transfected with an E-HA, M-HA, N-EGFP expressing plasmid, an EGFP-expressing plasmid, or cotransfected with an E-HA expressing plasmid and plasmids expressing SARS-CoV-2 M, N, S, or the HSV-1 γ_1_ 34.5 protein, respectively. Single transfections were done using 500 ng per well and cotransfections were performed using 1 μg per well (500 ng per plasmid). The cells were harvested at 24 h posttransfection and equal amounts of proteins were analyzed for expression of E-HA, S-HA, M-HA, γ_1_ 34.5 (Flag-tagged), or EGFP (control protein EGFP and N fused to EGFP). B-actin served as a loading control. Arrows indicate the E and M proteins that are both tagged with an HA epitope.

Both p-eIF-2α and LC3 lipidation could prevent E protein accumulation. To test this, we determined if γ_1_ 34.5 protein expression impacted E expression. HEK-293 cells were cotransfected with vectors expressing E and γ_1_ 34.5. Cells cotransfected with the E-expressing plasmid and a plasmid expressing EGFP, or cells transfected with the individual plasmids served as controls. The cells were harvested at 48 h posttransfection and E protein accumulation was assessed by analyzing equal amounts of proteins by Western blot. The levels of E protein were lower when coexpressed with EGFP compared with E alone, most likely because of competition of the two plasmids for transport into the nucleus, gene transcription and protein translation. However, when E was coexpressed with the γ_1_ 34.5 protein, accumulation of E protein was strongly enhanced (Fig. 3A). Expression of γ_1_ 34.5 protein did not affect N protein accumulation to the same extent as E protein accumulation (Fig. 3B). These data suggest that stress responses activated following E expression negatively impacted E accumulation; however, HSV-1 γ_1_ 34.5 protein could reverse these effects.

Considering that the activation of signaling responses following E expression may have deleterious effects on the host, SARS-CoV-2 must control E functions to ensure optimal virus replication (18, 19). Thus, we sought to determine if any SARS-CoV-2 proteins could reverse E effects allowing for E protein accumulation. We chose to analyze the effects of other virion proteins that may impact E functions through interactions. HEK-293 cells were cotransfected with plasmids expressing the E protein and either the M, N, or S proteins of SARS-CoV-2. HEK-293 cells were also cotransfected with plasmids expressing the E protein and either the EGFP or the HSV-1γ_1_ 34.5 protein to serve as negative and positive controls, respectively. As shown in Fig. 3C, both N and M proteins prevented p-eIF-2α accumulation due to E expression and enhanced E protein accumulation. Unlike E, neither N nor M protein expression caused eIF-2α phosphorylation (Fig. 3D). The S protein did not prevent p-eIF-2α accumulation and did not support E protein accumulation. Nevertheless, the γ_1_ 34.5 protein was more effective than the M and N proteins of SARS-CoV-2 in the accumulation of E protein. We also assessed the impact of different proteolytic machineries on E protein accumulation and found no significant effect (supplemental data and Fig. S1) negatively impacts its own accumulation, but this effect is reversed by proteins from SARS-CoV-2 or by heterologous viruses that appear to counterbalance E effects.

### SARS-CoV-2 E homologs and E oligomerization mutants trigger phosphorylation of eIF-2α.

**FIG 4:**
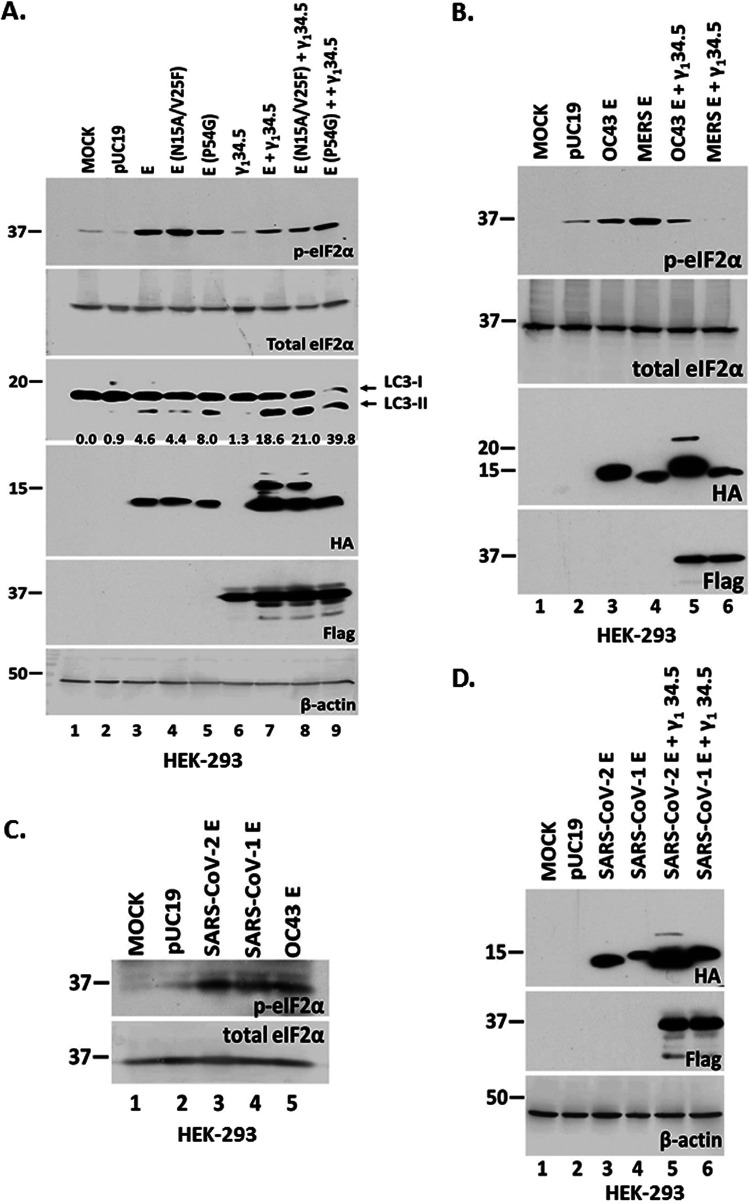
Homologs of E or oligomerization mutants do not abrogate phosphorylation of eIF-2α. (A) HEK-293 cells were transfected with plasmids expressing either wild-type E or various E oligomerization mutants. In addition, HEK-293 cells were transfected with a γ_1_ 34.5-expressing plasmid, or cotransfected with various E forms and a γ_1_ 34.5-expressing plasmid. The cells were harvested at 48 h posttransfection and equal amounts of proteins were analyzed for p-eIF-2α, total eIF-2α, E-HA, LC3, or γ_1_ 34.5 expression (Flag tagged). The ratio of LC3-II to LC3-I is also shown. B-actin served as a loading control. (B–D) Plasmids encoding E protein from different CoVs, including SARS-CoV, MERS-CoV, and HCoV-OC43 were transfected in HEK-293 cells or cotransfected with a γ_1_ 34.5-expressing plasmid. The cells were harvested at 48 h posttransfection and equal amounts of proteins were analyzed for p-eIF-2α, total eIF-2a, E-HA, and γ_1_ 34.5 expression (Flag-tagged). B-actin served as a loading control.

In the next series of experiments, we determined whether specific E mutants could decrease the E protein-induce ER stress responses. We inserted two point mutations in the transmembrane domain of the E protein, asparagine (N) at position 15 was converted to alanine (A) and the valine (V) at position 25 was converted to phenylalanine (F) (N15A/V25F). These mutations are known to reduce E oligomerization in SARS-CoV-1 to some extent (17). The N15A mutation reduces pentamerization of E, while V25F reduces higher order oligomers. As shown in Fig. 4A, the E N15A/V25F-expressing cells accrued similar levels of p-eIF-2α as cells expressing wild-type E (compare lane 4 to lane 3), suggesting that reduced E oligomerization does not reduce ER stress responses triggered by E. This level of p-eIF-2α was again reversed by γ_1_ 34.5 protein (compare lane 8 to lane 4). We also tested a mutant of E in which the conserved proline at position 54 was changed to a glycine (E-P54G). P54 is located within the cytoplasmic domain at the turn of a β-coil-β motif and likely affects E topology. E-P54G also triggered p-eIF-2α that was partially reversed by γ_1_ 34.5 protein (compare lane 5 to lane 3, and lane 9 to lane 5). LC3 lipidation was triggered by the unmodified E protein and all mutants tested, albeit to a greater extent by E-P54G. We conclude that disruption of E pentamerization or oligomerization does not reduce ER stress responses triggered by E expression.

We next sought to determine whether the E protein of SARS-CoV, MERS-CoV, and HCoV-OC43 trigger similar responses as SARS-CoV-2 E. Cells were cotransfected with E-expressing and γ_1_ 34.5 -expressing plasmids as described above. Like SARS-CoV-2 E protein, SARS-CoV, MERS-CoV, and HCoV-OC43 E homologs induced phosphorylation of eIF-2α (Fig. 4B-C). In each case the HSV-1 γ_1_ 34.5 protein blocked phosphorylation of eIF-2α (Fig. 4B) and enabled E protein accumulation (Fig. 4B and D). We conclude that E proteins from other pathogenic coronaviruses and E oligomerization mutants were able to induce ER stress responses that result in eIF-2α phosphorylation.

### The γ_1_ 34.5 protein of HSV-1 reverses the translational shutoff imposed by SARS-CoV-2 and restricts virus infection.

**FIG 5:**
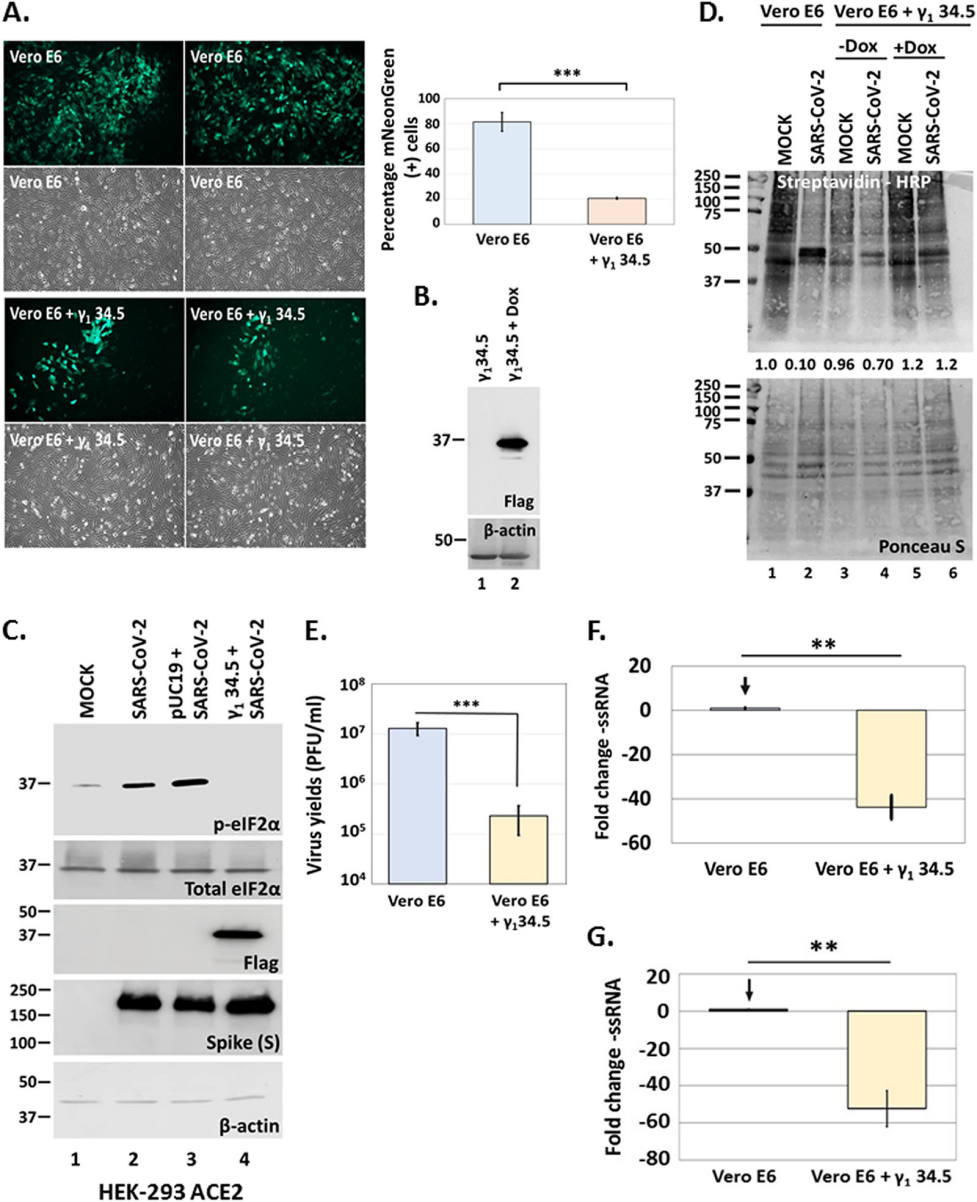
HSV-1 γ_1_ 34.5 inhibits SARS-CoV-2 infection. (A) Vero E6 + γ_1_ 34.5 cells were treated with doxycycline (5 μg/mL for 48 h) to induce γ_1_ 34.5 expression. Induced cells along with parental Vero E6 cells were infected with icSARS-CoV-2-mNG (10^−4^ PFU/cell). Images were captured at 24 h postinfection using an Olympus microscope. A quantification of mNeonGreen-positive cells in control versus Vero E6 + γ_1_ 34.5 cells is depicted. (B) Expression of γ_1_ 34.5-Flag protein following doxycycline treatment (20 μg/mL) of Vero E6 + γ_1_ 34.5 cells for 48 h. (C) HEK-293 ACE2-expressing cells were transfected with either γ_1_34.5 (Flag-tagged) expressing plasmid or the control plasmid pUC19. At 48 h posttransfection the cells were infected with SARS-CoV-2 (10 PFU/cell). The cells were harvested at 18 h postinfection and equal amounts of cell lysates were analyzed for p-eIF-2α, total eIF-2α, γ_1_ 34.5 (Flag), Spike (S), and β-actin. (D) Vero E6 and Vero E6 + γ_1_ 34.5, either untreated or treated with doxycycline (20 μg/mL) to induce γ_1_34.5 expression, were infected with SARS-CoV-2 (10^−4^ PFU/cell). At 34 h postinfection the cells were starved for 3 h in RPMI Medium 1640 without l-methionine (Thermo-Fisher) and subsequently incubated with medium supplemented with Click-iT AHA (l-azidohomoalanine) reagent (Invitrogen) for 2 h. Cells were lysed in a solution containing 1% SDS in 50 mM Tris-HCl, pH 8.0, and labeled proteins were reacted with biotin-alkyne (PEG4 carboxamide-propargyl biotin) in a Click-chemistry reaction according to manufacturer’s instructions using the Click-iT Protein Reaction Buffer kit (Invitrogen). Biotinylated proteins were analyzed in a denaturing polyacrylamide gel and detected with streptavidin-HRP. Both a Ponceau S staining of the membranes and the reaction of HRP with 4-chloro-1-naphthol supplemented with hydrogen peroxide are depicted. Quantification of total protein signal per sample relative to the signal of total proteins from uninfected cells, after normalization to the respective signal from Ponceau S staining is depicted. (E) Infections were performed with SARS-CoV-2 (10^−4^ PFU/cell) in replicate cultures of Vero E6 or doxycycline-treated (20 μg/mL) Vero E6 + γ_1_ 34.5 cells. The cells were harvested at 24 h postinfection and intracellular progeny virus was quantified by plaque assays in Vero E6 cells. (F–G) Infections were performed with either the wild type (panel F) or the reporter virus (panel G) as in panel E, in replicate cultures. Cells were harvested at 24 h postinfection and the –ssRNA was quantified by real-time PCR analysis.

Considering that HSV-1 γ_1_ 34.5 protein could antagonize E functions important for virus replication such as translational shutoff, we determined the effect of γ_1_ 34.5 on SARS-CoV-2 infection. We developed a Vero E6 cell line expressing γ_1_ 34.5 protein under a tetracycline inducible promoter (Tet ON) from an integrated lentiviral vector. At 48 h after inducing γ_1_ 34.5 expression, the Vero E6 + γ_1_ 34.5 cell line was infected with the reporter virus icSARS-CoV-2-mNG (10^−4^ PFU/cell) and mNeonGreen (mNG) expression was compared with infected Vero E6 cells. Expression of HSV-1 γ_1_ 34.5 protein resulted in fewer cells (~20%) that were positive for mNG compared with the control cells (~80%) (Fig. 5A). Expression of γ_1_ 34.5 protein at 48 h following induction with doxycycline was confirmed (Fig. 5B). We also found that infection with either the wild-type virus (Fig. 7A, C) or the reporter virus (Fig. 7B) triggered phosphorylation of eIF-2α in Vero E6 cells, but not in γ_1_ 34.5 -expressing cells. Similar results were obtained in HEK-293 ACE2-expressing cells where SARS-CoV-2 triggered eIF-2α phosphorylation that was inhibited by ectopic expression of γ_1_ 34.5 protein (Fig. 5C), even though infections were performed at high multiplicity of infection (10 PFU/cell). At high multiplicity of infection the levels of Spike protein were comparable in the presence or absence of γ_1_ 34.5 protein, due to detection of virion Spike present in the inoculum.

We then asked if phosphorylation of eIF-2α leads to host translational shutoff during SARS-CoV-2 infection, and whether it could be inhibited by γ_1_ 34.5 protein expression. The Vero E6 + γ_1_ 34.5 cell line that was either uninduced or induced to express γ_1_ 34.5 protein and parental cells were infected with SARS-CoV-2 (10^−4^ PFU/cell). Cells were starved of methionine for 3 h starting at 34 h postinfection followed by labeling with the amino acid analog of methionine L-azido homoalanine. Click-chemistry was used to detect the labeled proteins. As shown in Fig. 5D, SARS-CoV-2 infection caused translational shutoff (lane 2 compared with lane 1) that was inhibited in the presence of γ_1_ 34.5 (lane 6 compare with lane 2). An intermediate phenotype was observed in infected, uninduced Vero E6 + γ_1_ 34.5 cells (lane 4 compare with lanes 2 and 6), perhaps due to some leakiness of γ_1_ 34.5 protein expression. Staining with Ponceau S of total proteins served as a loading control.

To quantify the effect of γ_1_ 34.5 on SARS-CoV-2 infection, the Vero E6 + γ_1_ 34.5 cell line that was induced to express γ_1_ 34.5 protein and Vero E6 cells were infected with SARS-CoV-2 (10^−4^ PFU/cell) and progeny virus production was quantified at 24 h postinfection by plaque assays. The presence of γ_1_ 34.5 protein caused an approximate 100-fold reduction in infectious virus production at 24 h postinfection (Fig. 5E). In a similar assay, we compared the amounts of the negative-strand RNA (-ssRNA) of the virus in the γ_1_ 34.5-expressing cell line versus parental cells. We found that the presence of γ_1_ 34.5 protein caused more than 40-fold and more than 50-fold decrease in the -ssRNA of the WT virus and the reporter virus, respectively (Fig. 5F-G). Overall, γ_1_ 34.5 was able to inhibit the translational shutoff imposed during SARS-CoV-2 infection, which resulted in a decrease in virus replication and progeny virus production.

### Inhibition of PERK prevented eIF-2α phosphorylation and had a negative effect on SARS-CoV-2 infection.

**FIG 6:**
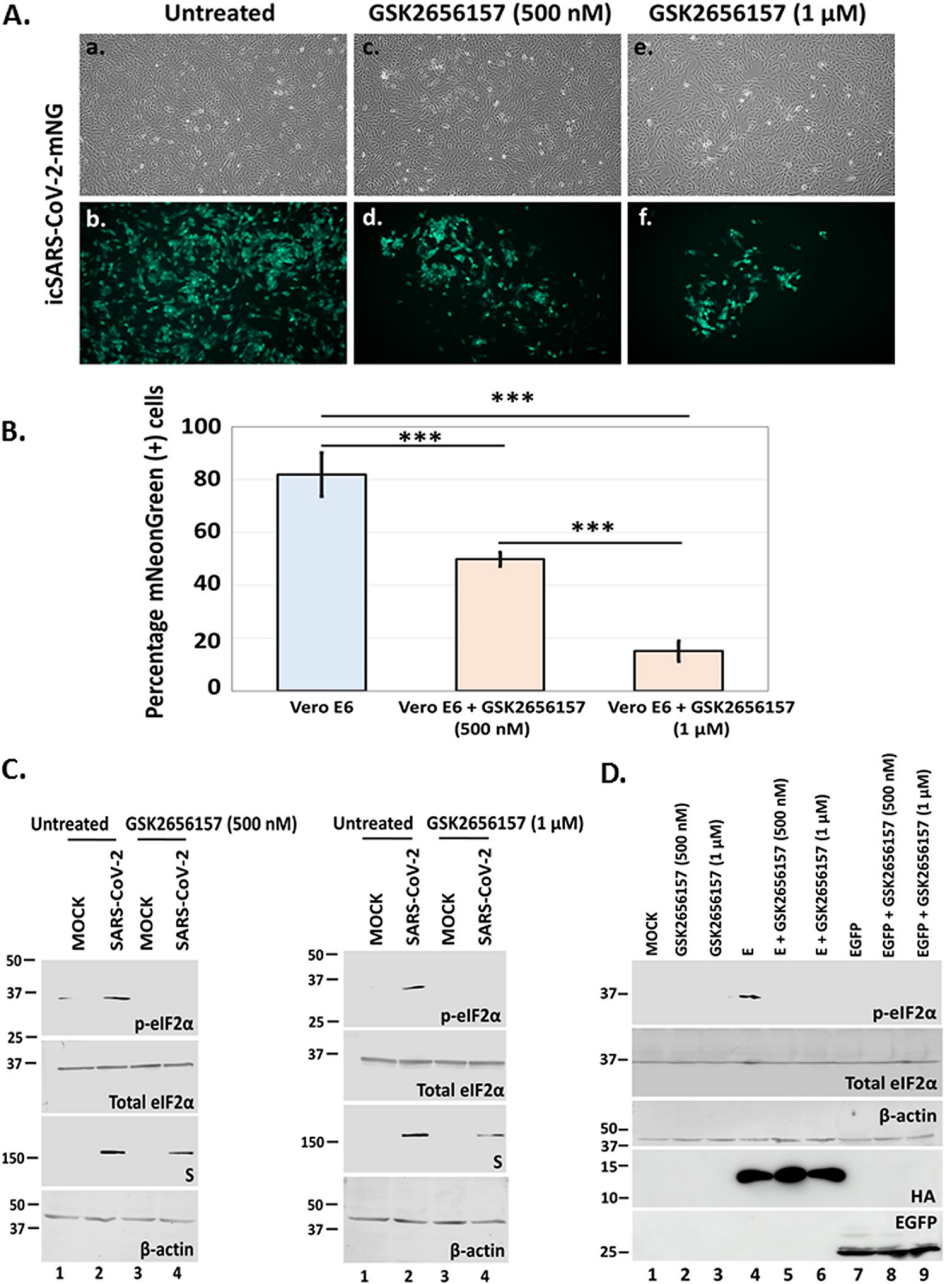
A PERK inhibitor inhibits SARS-CoV-2 infection. (A) Vero E6 cells were infected with icSARS-CoV-2-mNG (10^−4^ PFU/cell). Cells were then either left untreated or treated at 1 h postinfection with GSK2656517 (Sigma-Aldrich) at a dose of either 500 nM or 1 μM. Images were captured at 24 h postinfection using an Olympus microscope. (B) A quantification of mNG –expressing cells over total number of cells from panel A is depicted. (C) Vero-E6 cells treated as in panel A were harvested at 24 h postinfection and equal amounts of lysates were analyzed for expression of p-eIF-2α, total eIF-2α, S, or β-actin. (D) HEK293 cells were untransfected or transfected with plasmids expressing either E-HA or EGFP. At 6 h posttransfection cells were either left untreated or treated with GSK2656517 at a dose of either 500 nM or 1 μM. At 24 h posttransfection cells were harvested and equal amounts of lysates were analyzed for expression of p-eIF-2α, total eIF-2α, HA, EGFP, or B-actin.

Expression of γ_1_ 34.5 protein blocked effectively eIF-2α phosphorylation and inhibited SARS-CoV-2 infection. To exclude the possibility that this effect was due to an undocumented function of γ_1_ 34.5 protein, we chose a pharmacological approach to inhibit PERK that usually mediates eIF-2α phosphorylation due to stress responses. We observed that in the presence of a PERK inhibitor fewer cells expressed mNG as opposed to untreated cells, following infection with the reporter virus (Fig. 6A–B). The inhibitory effect of the drug was dose-dependent (Fig. 6A-B). Also, the PERK inhibitor prevented eIF-2α phosphorylation during SARS-CoV-2 infection, in a dose-dependent manner, and caused a decrease in S protein accumulation (Fig. 6C). Finally, the PERK inhibitor blocked eIF-2α phosphorylation caused by overexpression of SARS-CoV-2 E protein (Fig. 6D). Overall, chemical inhibition of PERK prevented phosphorylation of eIF-2α and inhibited SARS-CoV-2 infection.

### γ_1_ 34.5 protein alters autophagic responses during SARS-CoV-2 infection.

**FIG 7:**
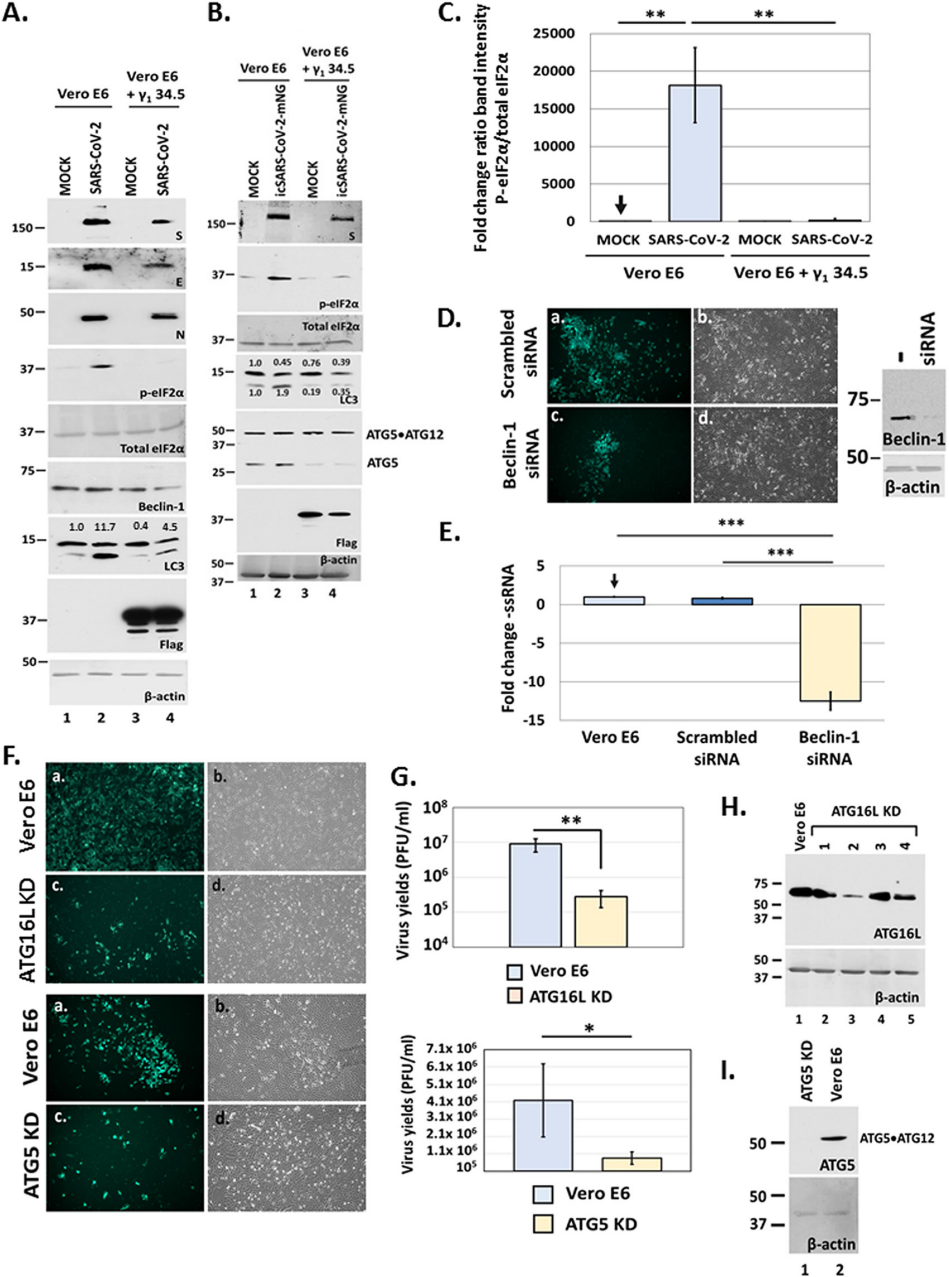
Effect of γ_1_ 34.5 protein on autophagy during SARS-CoV-2 infection. (A–B) Replicate cultures of Vero E6 and doxycycline-induced Vero E6 + γ_1_ 34.5 cells were infected with either the SARS-CoV-2 USA-WA1/2020 (panel A) or the reporter virus icSARS-CoV-2-mNG (panel B) (10^−4^ PFU/cell). Cells were harvested at 36 h postinfection and equal amounts of proteins were analyzed for p-eIF-2α, total eIF-2α, LC3, γ_1_ 34.5 (Flag-tagged), ATG5, Beclin-1, S, E, N protein expression, and β-actin. Numbers represent ratio of LC3-II/LC3-I (panel A) and a quantification of LC3-I and LC3-II (panel B). (C) Quantification of band intensity of p-eIF-2α versus total eIF-2α relative to uninfected, Vero E6 cells from at least three independent experiments is depicted. (D) Vero-E6 cells were transfected at 50% confluence with either a control (scrambled) siRNA (Santa Cruz; sc-37007) or Beclin 1 siRNA (Santa Cruz; sc-29797) using Lipofectamine 3000 according to manufacturer’s instructions (Invitrogen). Both siRNAs were used at a 300 nM concentration and the cells were transfected for 72 h before infection. Efficiency of Beclin-1 depletion is depicted. Vero E6 cells treated with either the scrambled siRNA, or the Beclin-1 siRNA as above were infected with icSARS-CoV-2-mNG (10^−4^ PFU/cell). Images were captured at 24 h postinfection using an Olympus microscope. (E) Vero E6 cells treated with Beclin-1 and scrambled siRNA as above were infected with wild type SARS-CoV-2 (10^−4^ PFU/cell). The cells were harvested at 24 h postinfection followed by total RNA extraction. Quantification of the negative-sense RNA was done by RT-qPCR analysis. (F) Vero E6, ATG16L KD and ATG5 KD derivatives were infected with icSARS-CoV-2-mNG, as above and images were captured at 24 h postinfection. (G) Infections of Vero E6 cells and ATG16L KD or ATG5 KD derivatives were performed with the wild-type SARS-CoV-2. Samples were harvested at 24 h postinfection and quantification of progeny virus production was performed by plaque assays. All values were derived after analyzing samples from three independent experiments. *, *P* ≤ 0.05; **, *P* ≤ 0.01; ***, *P* ≤ 0.001. (H–I) Efficiency of ATG16L or ATG5 depletion using specific shRNAs (Sigma) expressed from integrated lentiviral vectors is depicted. To deplete ATG16L four different cell lines were established using four different shRNAs (1–4). The cell line expressing shRNA number 2 was selected as depletion of ATG16L was more efficient.

During SARS-CoV-2 infection autophagy appears to supplement the viral membrane factories with structural components and metabolites required for their formation, expansion, and virus replication (44–46). While γ_1_ 34.5 is known to interfere with autophagy by binding to Beclin-1, we observed that coexpression of γ_1_ 34.5 protein with E did not reduce, but rather enhanced LC3 lipidation (Fig. 2D). LC3 lipidation was also induced during SARS-CoV-2 infection, however in the presence of γ_1_ 34.5, the levels of both nonlipidated and lipidated LC3 were reduced (panel 7A, compare lane 4 to lane 2 and panel 7B, compare lane 4 with lane 2).We also noticed a decrease in the amounts of S, E, N proteins, Beclin-1 and ATG5 in infected γ_1_ 34.5 -expressing cells, although the levels of the ATG5/ATG12 complex remained unaltered. These data indicate that γ_1_34.5 disrupted autophagic responses during SARS-CoV-2 infection, causing a reduction in viral infection. Consistent with our findings in Fig. 5, SARS-CoV-2 infection caused phosphorylation of eIF-2α in Vero E6, but not in the presence of γ_1_ 34.5 (Fig. 7A-7B). Quantification of p-eIF-2α/total eIF-2α protein band intensity of the different samples compared with uninfected Vero E6 cells is depicted in Fig. 7C.

The γ_1_ 34.5 protein is known to combat autophagy during HSV-1 infection through both a direct mechanism, by interacting with Beclin-1, and an indirect mechanism, by inhibiting PKR-induced phosphorylation of eIF-2α. To assess the impact of Beclin-1 on SARS-CoV-2 infection we depleted cells of Beclin-1 using a specific siRNA followed by infection with either the reporter virus (Fig. 7D) or wild-type virus (Fig. 7E). Depletion of Beclin-1 resulted in fewer mNG-expressing cells (Fig. 7D) and an inhibition in SARS-CoV-2 infection that corresponded to approximately 14-fold decrease in the amounts of negative-sense RNA compared with untreated or scrambled siRNA -treated cells (Fig. 7E). Additionally, we performed infections in cells depleted of ATG16L or ATG5, two critical factors for synthesis of the autophagosome precursor. Infection of ATG16L KD or ATG5 KD cells with the reporter virus resulted in fewer cells expressing mNG compared with parental cells (Fig. 7F). Also, ATG16L KD and ATG5 KD cells displayed reduced SARS-CoV-2 progeny virus production by approximately 100-fold and 10-fold, respectively (Fig. 7G). The efficiency of ATG16L and ATG5 depletion is depicted in Fig. 7H and 7I, respectively. These data suggest that early autophagy events are essential during SARS-CoV-2 infection.

**FIG 8:**
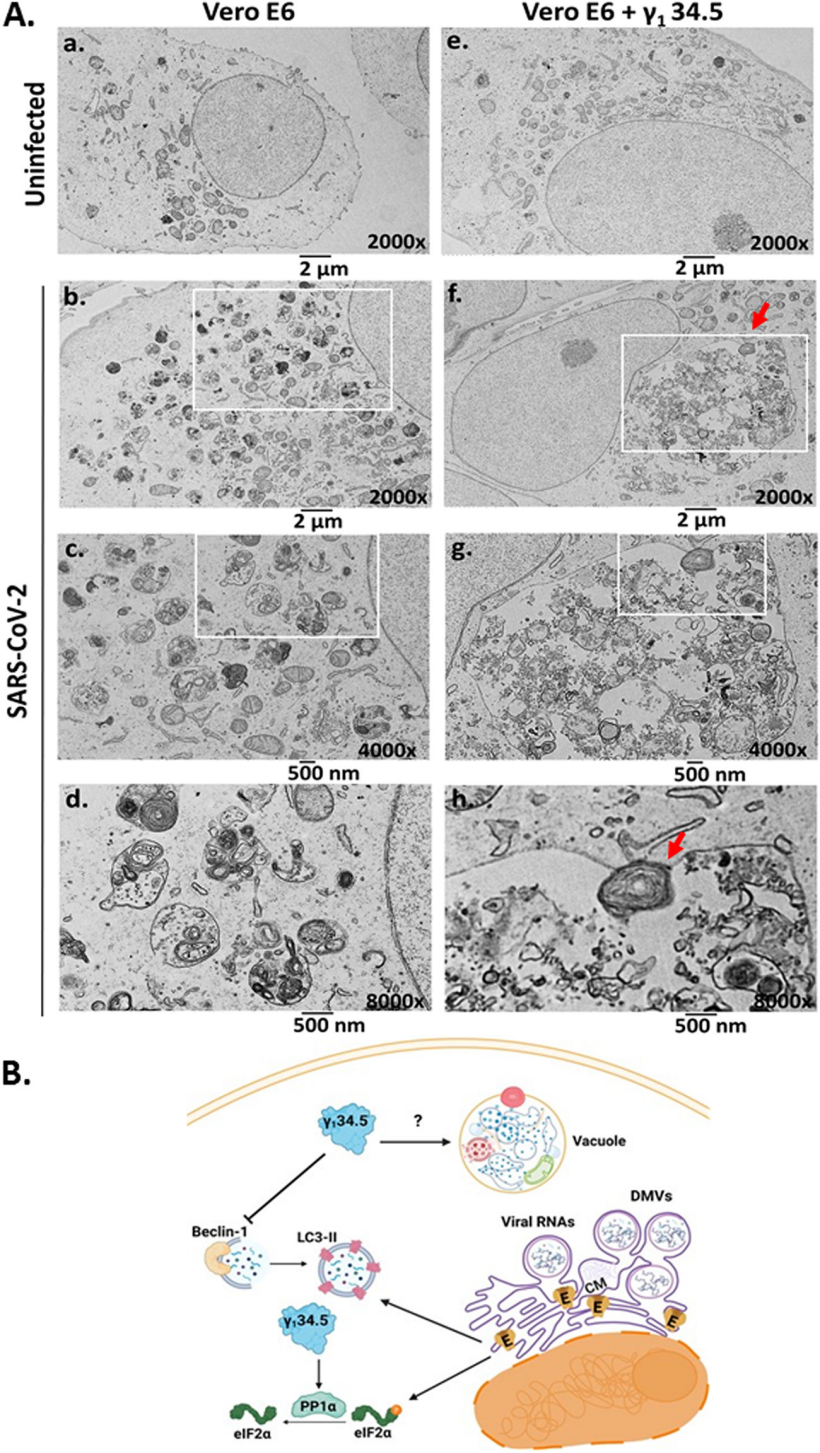
Aberrant vacuolar structures in the cytoplasm of SARS-CoV-2 infected γ_1_ 34.5 – expressing cells. (A) Replicate cultures of Vero E6 and doxycycline-induced Vero E6 + γ_1_ 34.5 cells seeded on coverslips were infected with SARS-CoV-2 USA-WA1/2020 (10^−4^ PFU/cell). The cells were fixed with 2% glutaraldehyde at 42 h postinfection and processed for TEM analysis, as detailed in Materials and Methods. At least 50 cells were analyzed per sample. Representative images are depicted. (B) Model summarizing the mechanism of interference of SARS-CoV-2 replication by the HSV-1 γ_1_ 34.5 protein. SARS-CoV-2 infection causes extensive reorganization of the ER/ERGIC compartments that leads to formation of viral membrane factories where the virus replicates. It also imposes a translational shutoff that offers an advantage to viral over host genes for expression, while suppressing host defense gene expression. E protein could contribute to virus replication by facilitating membrane rearrangements through activation of autophagy that supports the growth of the viral factories. E along with other viral proteins could be responsible for the translational shutoff during SARS-CoV-2 infection. γ_1_ 34.5 protein could disrupt autophagy activated during SARS-CoV-2 infection by binding to Beclin-1 and/or through other mechanisms. Also, γ_1_ 34.5 protein suppresses host translational shutoff during SARS-CoV-2 infection. Both effects could result in inhibition of SARS-CoV-2 replication. Autophagic vacuoles with abnormal size and morphology observed in SARS-CoV-2 infected γ_1_ 34.5 -expressing cells could represent defects in the autophagolysosome pathway.

Finally, we determined if γ_1_ 34.5 expression could cause overt changes in the viral membrane factories through its effects on autophagy. For this, doxycycline-treated Vero E6 + γ_1_ 34.5 cells and parental cells that were or were not exposed to SARS-CoV-2 were processed for TEM analysis. Extensive membrane rearrangements and aberrant vesicular structures were observed in the cytoplasm of SARS-CoV-2 infected cells compared with uninfected cells (Fig. 8, compare panels B–D to panel a). However, infection of γ_1_ 34.5-expressing cells resulted in formation of oversized vacuoles that contained what appeared to be trapped cellular organelles, including mitochondria and endosomes undergoing degradation (Fig. 8, compare panels F–H to panel E). A potential engulfment or fusion event with an organelle resembling a lysosome has been marked with a red arrow (Fig. 8, panel H). We conclude that γ_1_ 34.5 expression during SARS-CoV-2 infection altered autophagic responses and caused formation of abnormal vacuoles with different organelles entrapped undergoing degradation.

## DISCUSSION

Our studies emanated from the observation that SARS-CoV-2 causes major rearrangements in the ER-Golgi membranes that form the viral membrane factories, where the virus replicates and virions assemble. Since E is a small transmembrane protein that oligomerizes in the ER and ERGIC, we hypothesized that it could disrupt the functions of these organelles (3–5, 7). Indeed, we observed that E protein caused LC3 lipidation, a hallmark of autophagy initiation, and phosphorylation of the translation initiation factor eIF-2α resulting in host translational shutoff. It is likely that E expression activates ER-stress responses, including the unfolded protein response that results in PERK activation, which phosphorylates eIF-2α (42, 43, 47). Disruption of ER homeostasis could subsequently lead to autophagy activation.

Coronaviruses are known to impose host translational shutoff during the early stages of infection to prevent the infected host from synthesizing new proteins while translation of viral mRNAs is not affected (48–60). Phosphorylation of eIF-2α has been reported during infection by SARS-CoV, SARS-CoV-2, transmissible gastroenteritis virus (TGEV) and other CoVs and both kinases PERK and PKR appear to participate in this process (61–67). PERK could be activated following disruption of ER homeostasis by viral proteins accumulating in the ER (68). For example, E could disrupt ER Ca^2+^ homeostasis through its viroporin function. PKR could be activated following viral RNA sensing. Ectopic expression of several SARS-CoV proteins, including S and ORF3a triggers p-eIF-2α due to disruption of ER homeostasis (69–71). Inhibition of host cell translation by coronaviruses that does not involve phosphorylation of eIF-2α has also been reported and occurs through the coronavirus Nsp1 protein. Nsp1 has evolved to target multiple steps in the host cell mRNA biogenesis pathway, including its binding to the 40S ribosomal subunit and obstruction of the mRNA entry tunnel, and its ability to block mRNA export from the nucleus. We observed that ectopic expression of E protein had deleterious effects on the host cell that impacted E protein accumulation. It is not uncommon for viruses to develop mechanisms to regulate and harness the activity of proteins that trigger deleterious responses to ensure optimal replication. To test this, we coexpressed E with either other SARS-CoV-2 proteins that are known to interact with E, or with proteins from heterologous viruses that can evade host translational shutoff and autophagy. We discovered that the γ_1_ 34.5 protein of HSV-1 inhibited phosphorylation of eIF-2α triggered by E expression and allowed for E protein accumulation. The mechanism by which γ_1_ 34.5 protein prevents accumulation of p-eIF-2α has been previously described. During HSV-1 infection, PKR is activated and phosphorylates eIF-2α. HSV-1 γ_1_ 34.5 recruits the PP1α phosphatase to dephosphorylate eIF-2α (26, 27, 31, 32). While γ_1_ 34.5 protein inhibited accumulation of p-eIF-2α in E-expressing cells, it did not inhibit LC3 lipidation. The γ_1_ 34.5 protein can inhibit autophagy by binding to the phagophore nucleation factor Beclin-1 and by inhibiting different pattern recognition receptors and downstream effectors like the TANK-binding kinase 1 (TBK1) (30, 72, 73). Recent studies demonstrated that depletion of Beclin-1 had little effect on LC3 lipidation, but it played a critical role during autophagosome formation and macromolecule degradation through the autophagy pathway (74). These observations may explain why LC3 lipidation occurs when E protein is coexpressed with γ_1_ 34.5.

Besides γ_1_ 34.5, we found that the M and N proteins of SARS-CoV-2 prevented accumulation of p-eIF-2α in E-expressing cells, while S exacerbated accumulation of p-eIF-2α. Both M and N interact with E during virion assembly (1). It is likely that this binding alters the localization of E, its oligomerization status, or its potential binding with host factors, which alters its propensity to cause ER stress responses. Alternatively, the immunoevasion properties of M and N could reverse the translation inhibition imposed by E (75, 76). On the other hand, ectopic expression of S is known to trigger phosphorylation of eIF-2α, and in the presence of E such an effect was exacerbated (69). Consistent with this, we have observed increased LC3 lipidation when E and S were coexpressed ectopically (data not shown).

The properties and functions of the E proteins are generally conserved among beta coronaviruses, as the E proteins of SARS-CoV, MERS-CoV, and HCoV-OC43 were found to trigger similar responses as SARS-CoV-2 E (77). Mutations that abrogated the ion channel function of E or decreased E oligomerization did not suppress p-eIF-2α accumulation. Perhaps the intracellular localization of E and its interactors are sufficient to trigger ER stress responses leading to eIF-2α phosphorylation.

An interesting observation was that SARS-CoV-2 displayed decreased replication, viral protein expression, and progeny virus production in γ_1_ 34.5-expressing cells (see model Fig. 7B). One explanation is that by preventing host translational shutoff, γ_1_ 34.5 decreased the efficiency of viral gene expression and enabled expression of host defense genes that are known to combat SARS-CoV-2 infection (48). Considering that host translational shutoff during SARS-CoV-2 infection is the result of both eIF-2α phosphorylation and reduced accessibility of mRNAs to ribosomes, γ_1_ 34.5 likely inhibited the one, but not the other mechanism. However, this amount of host protein synthesis appears to be sufficient to obstruct the infection. Consistently, treatment with a PERK inhibitor that prevented phosphorylation of eIF-2α had an inhibitory effect on SARS-CoV-2 infection. An additional possibility is that γ_1_ 34.5 disrupted autophagy pathways utilized by SARS-CoV-2. Coronaviruses are known to exploit autophagosome formation to support DMV biogenesis, while stalling lysosome fusion to evade autophagy-mediated degradation. Transient depletion of Beclin-1, which functions as a scaffold in forming a multiprotein assembly during autophagy initiation and nucleation, and is a known target of γ_1_ 34.5, obstructed the infection. In addition, depletion of ATG16L, an integral part of the complex involved in LC3 lipidation that is essential for autophagosome formation and expansion, had a negative effect on SARS-CoV-2 infection. A similar negative effect was observed when cells were depleted of ATG5. ATG5 along with ATG12 form an E3-like enzyme that lipidates ATG8 family proteins, including the LC3 member, facilitating phagophore elongation. Thus, it appears that LC3 lipidation is important for SARS-CoV-2 replication. Another striking observation was that infection of γ_1_ 34.5-expressing cells with SARS-CoV-2 led to formation of enormous vacuoles that were almost half the size of the nucleus containing what appeared to be various organelles undergoing degradation. This abnormal phenotype of vacuoles may be the result of either defective autophagy or defective proteolysis. While SARS-CoV-2 through ORF3a can inhibit fusion of autophagosomes with lysosomes and decrease lysosomal activity by increasing lysosomal pH, this was not sufficient to yield the abnormal vacuole phenotype, and γ_1_ 34.5 was required to sensitize the cells by altering early autophagy events.

Overall, these studies provide novel evidence that ectopic expression of E causes adverse effects on the host cell. These effects were found to be antagonized by γ_1_ 34.5, a protein from a heterologous virus. This led us to discover that the activity of E is likely regulated during SARS-CoV-2 infection by other viral proteins to ensure optimal virus production. Finally, we demonstrated that pathways inhibited by γ_1_ 34.5 are required for optimal SARS-CoV-2 growth, therefore these pathways could be considered novel antiviral targets.

## MATERIALS AND METHODS

### Cells and viruses.

The Caco-2 (human colorectal adenocarcinoma), HEK-293 (human embryonic kidney epithelial cells), and Vero E6 (normal monkey kidney epithelial cells) were obtained through ATCC. The HEK-293 ACE2 and the A549 ACE2 cells (human lung adenocarcinoma), were obtained through BEI resources. The SARS-related coronavirus 2 isolate USA-WA1/2020 was obtained through BEI resources (NR-52281). The icSARS-CoV-2-mNG was obtained through the World Reference Center for Emerging Viruses and Arboviruses (“WRCEVA”) at the University of Texas Medical Branch at Galveston (“UTMB”).

### SARS-CoV-2 propagation and titration.

Vero E6 cells in 10 × 150 mm dishes were infected with either SARS-CoV-2 or icSARS-CoV-2-mNG. Culture supernatants were collected when extensive cytopathic effects were observed (72 to 96 h postinfection), floating cells and cellular debris were removed by low-speed centrifugation and virus yields were determined by serial dilution in Vero E6 cells. Virus spread was restricted using 1% methylcellulose.

### Development of a Vero E6 cell line expressing γ_1_ 34.5 under tetracycline inducible promoter from an integrated lentiviral vector.

A plasmid expressing the γ_1_34.5 ORF with a FLAG-tag was digested with HindIII and Xba I to extract only the FLAG-tagged γ_1_34.5. This fragment was then inserted into the pLenti-mCherry-Mango II x 24 plasmid (Addgene number 127587) digested with Nhe I and BamH I. HEK-293 cells were seeded in a 60 mm dish at 60% confluence and were cotransfected with the pLenti-mCherry-FLAG-γ_1_34.5 plasmid described above, the Gag-Pol-expressing plasmid, and the vesicular stomatitis virus G (VSV-G)-expressing plasmid at a ratio of 7:7:1 (5 μg total amount of DNA) using Lipofectamine 3000 (Invitrogen) according to the manufacturer’s instructions. At 48 h after transfection, the supernatant from the cultures was collected, filtered through a 0.45-μm-pore-size filter, and used to infect Vero-E6 cells, as described before (78, 79). Puromycin selection initiated at 24 h after exposure of cells to lentiviruses and continued until only resistant clones survived. Resistant cultures were then plated in a 6-well plate and exposed to doxycycline (5 μg/mL) for 48 h. After 48 h the cells were harvested in triple lysis solution and equal amounts of lysates were analyzed for expression of FLAG-γ_1_34.5 via immunoblot analysis. Cultures with the greatest expression of FLAG-γ_1_34.5 were then used for all further experiments.

### Plasmids and transfection assays.

The genes for the E proteins were synthesized by SynBio Technologies with HA-tags fused to the C terminus of each protein. The genes were expressed in the pcDNA3.1 (+) vector. The vectors expressing the SARS-CoV-2 M and S proteins (with C-terminal HA-tag) were obtained from Sino Biologicals. The pcDNA3.1(+)-N-eGFP-N plasmid, expressing N from SARS-CoV-2, was obtained from GenScript Biotech (Catalog number MC_0101137).

Transfections of HEK-293 cells, seeded in 12-well plates, were performed using Lipofectamine 3000 according to the manufacturer’s instructions. Unless stated otherwise, all transfections were done using 1 μg of DNA total for single transfections, or 1.5 μg total for cotransfections (750 ng per plasmid). Cells were harvested at 48 h posttransfection and equal amounts of protein were analyzed by immunoblot analysis.

### Western blot and antibodies.

The procedures for immunoblotting were described elsewhere (78, 80). All Western blots are representative of the results Briefly, cells were solubilized in triple-detergent buffer (50 mM Tris-HCl [pH 8], 150 mM NaCl, 0.1% sodium dodecyl sulfate, 1% Nonidet P-40, 0.5% sodium deoxycholate, 100 μg of phenylmethylsulfonyl fluoride mL − 1) supplemented with phosphatase inhibitors (10 mM NaF, 10 mM β-glycerophosphate, 0.1 mM sodium orthovanadate) and protease inhibitor cocktail (Sigma) and briefly sonicated. The protein concentration was determined with the aid of the Bio-Rad protein assay (Bio-Rad Laboratories). 10 to 40 micrograms of total proteins per sample were subjected to further analysis. The mouse monoclonal antibodies to p62/SQSTM1 (Cell Signaling), OPTN (Santa Cruz), and Beclin-1 (Santa Cruz) were used at a 1:1,000 dilution. The mouse monoclonal antibodies to HA-tag (Santa Cruz), GFP (Santa Cruz), ATG5 (Santa Cruz), ATG16 (Santa Cruz), β-actin (Sigma) and Flag-tag (Sigma; clone M2) were used in a 1:2,000 dilution. The rabbit polyclonal antibody against LC3-B (Novus Biological) was used in a 1:2,000 dilution. The rabbit monoclonal antibodies against phospho-eIF2α and total eIF2α (Cell Signaling Technology number 3597and number 5324, respectively) were used in a 1:1,000 dilution. The rabbit polyclonal antibody against SARS-CoV-2 spike and the rabbit monoclonal antibody against SARS-CoV-2 nucleocapsid were obtained through BEI resources and used at a 1:1,000 and 1:2000 dilution, respectively. The rabbit polyclonal antibody against the SARS-CoV-2 envelope protein was obtained through Cell Signaling Technology (number 74698) and used at a 1:500 dilution. Protein bands were visualized with 5-bromo-4-chloro-3-indolylphosphate (BCIP)-nitroblue tetrazolium (NBT) (VWR) or with enhanced-chemiluminescence (ECL) Western blotting detection reagents (Pierce) according to the manufacturer’s instructions.

### Monitoring of nascent protein synthesis.

Cells were uninfected or infected with SARS-CoV-2 for indicated times. Starvation of cells was done for 3 h by incubating with RPMI Medium 1640 without l-methionine (Thermo Fisher). Cells were then incubated with the same medium supplemented with the Click-IT-AHA (L-Azidohomoalanine) reagent (Invitrogen) for 2 h. Cells were lysed in a solution containing 1% SDS in 50 mM Tris-HCl, pH 8.0. Click-chemistry reaction for protein detection was performed using biotin alkyne (PEG4 carboxamide-propargyl biotin) according to manufacturer’s instructions using the Click-iT Protein Reaction Buffer kit (Invitrogen). Labeled proteins were separated by SDS-PAGE. The membrane containing the biotin alkyne labeled proteins was incubated in a solution containing 1% BSA with streptavadin-HRP (Invitrogen) for subsequent visualization.

### Detection of viral negative sense –ssRNA.

Cell lysates were collected in TRIzol reagent (Ambion) at indicated times postinfection. Total RNA was extracted via phenol-chloroform extraction method. Reverse-transcription PCR was then performed using LunaScript RT Master Mix kit (NEB) using a gene specific primer. This primer was used to specifically detect the negative-strand RNA from SARS-CoV-2, as described previously (81). The reverse transcription primer (1 μM final concentration), 5′-ACAGCACCCTAGCTTGGTAGCCGAACAACTGGACTTTATTGA -3′, contains IAC (internal amplification control) tag-2 and a part targeting the ORF1ab gene of SARS-CoV-2. Real-time PCR was then performed using SYBR green reagent (Invitrogen) according to the manufacturer’s recommendations in a 7500 fast real-time PCR system (Applied Biosystems). The forward primer, 5′- AGGTGTCTGCAATTCATAGC-3′ (743 to 762bp), and the reverse primer, 5′- ACAGCACCCTAGCTTGGTAG -3′ (IAC tag-2) (500nMfinal concentration), were used for amplification.

### Processing cells for transmission electron microscopy (TEM).

Cell monolayers on Thermanox plastic coverslips (13 mm) (Nunc) were fixed with 2% glutaraldehyde in 0.1 M sodium cacodylate buffer, pH 7.4, and washed two times with 0.1 M sodium cacodylate buffer. Samples were postfixed in 1% osmium tetroxide plus 1.5% potassium ferrocyanide in 0.1 M sodium cacodylate for 30 min at room temperature and rinsed 3 times with distilled water. Samples were dehydrated in a graded series of ethanol as follows: 50%, 70%, 80%, 95%, 100%, 100%. A drop of Embed 812 resin was applied to each coverslip and samples embedded on Thompson molds were polymerized overnight at 60°C. Coverslips were peeled from mold, blocks were trimmed and sectioned. Ultrathin sections contrasted with 3% uranyl acetate for 5 min and 3% Reynolds lead citrate for 5 min. Samples were viewed using a JEOL JEM-1400 TEM at 100KV and digital images acquired with an AMT digital camera.

### Statistical analysis.

The *P* values were calculated using a standard unpaired Student's *t* test with a *p* ≤0.05 considered significant. All statistical analyses were performed using at least three biological replicates.
