# Supplementary material for: SARS-CoV-2 Harnesses Host Translational Shutoff and Autophagy To Optimize Virus Yields: the Role of the Envelope (E) Protein
Source: Microbiol Spectr. 2023 Jan 9;11(1):e03707-22. doi: 10.1128/spectrum.03707-22 (PMC9927098; doi:10.1128/spectrum.03707-22)
Supplement: Supplemental file 1 — Supplemental material. Download spectrum.03707-22-s0001.pdf, PDF file, 0.5 MB [file spectrum.03707-22-s0001.pdf]

Supplemental figure legends

Figure S1: Caspase-dependent, but proteasome and autophagy–independent down-modulation of SARS-CoV-2 E. A. HEK-293 cells were either left untransfected, or transfected with an E-HA expressing plasmid, or a control plasmid. The pan-caspase inhibitor Z-VAD-FMK (10 μM) and the proteasome inhibitor MG132 (10 μM) were added to the cultures for 14 h. The cells were harvested at 48 h post-transfection and equal amounts of proteins were analyzed for E protein expression. **B. C. D.** Single and double transfections were performed as in Figure. 3. Z-VAD-FMK (10 μM) and the autophagy inhibitor bafilomycin A1 (10 μM) were added to the cultures for 14 h. The cells were harvested at 48 h post-transfection and equal amounts of proteins were analyzed for HA-tagged E. β-actin served as a loading control.

Supplemental data

Inhibition of different proteolytic machineries had only a minor effect on E protein accumulation. A series of control experiments were performed to further confirm that decreased E accumulation was due to translational shutoff and not due to protein degradation. HEK-293 cells were transfected with a vector expressing E and at 24 h post-transfection, cells were treated with either Z-VAD-FMK, a pan-caspase inhibitor, or with MG132, a proteasome inhibitor. The cells were harvested at 48 h post-transfection and the levels of E protein were determined in equal amounts of cell lysates. As shown in Figure S1A-B, treatment with Z-VAD-FMK moderately enhanced E protein accumulation, but MG132 did not. We also determined if the effect of γ_1 34.5 on E accumulation was through the inhibition of caspases. For this, cells co-transfected with E and γ_1 34.5-expressing plasmids or E-expressing plasmid alone were treated with Z-VAD-FMK or left untreated. The results in Figure S1C showed that Z-VAD-FMK resulted in less E protein

accumulation than γ_1 34.5, indicating that the effect of Z-VAD-FMK and γ_1 34.5 on E accumulation was additive. A similar analysis was performed using bafilomycin A1, a vacuolar H^+ ATPase (V-ATPase) inhibitor that inhibits autophagy. Bafilomycin A1 had only a small positive effect on E protein accumulation, primarily when γ_1 34.5 was co-expressed (Figure S1D). Taken together, these data suggest that γ_1 34.5 expression results in E protein accumulation by a mechanism that is independent of caspases, proteasome, and autophagy inhibition and is consistent with inhibition of translational shutoff.

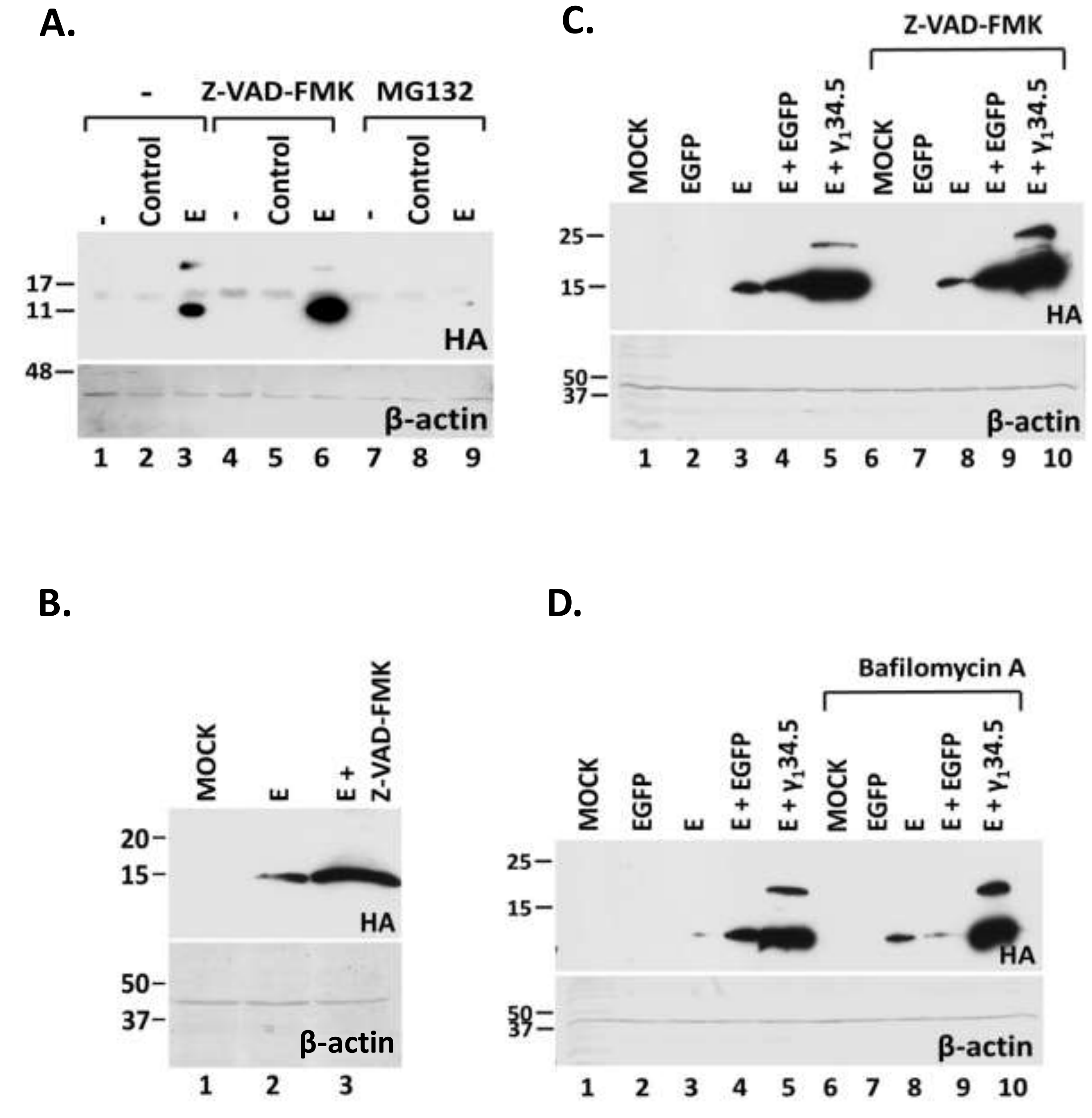

Supplemental Figure 1
